# Imiquimod-induced bullous pemphigoid: A case report

**DOI:** 10.1177/2050313X231164222

**Published:** 2023-03-30

**Authors:** Heidi Oi-Yee Li, Michael Aw, Steven J Glassman

**Affiliations:** 1 Division of Dermatology, University of Ottawa and The Ottawa Hospital, Ottawa, ON, Canada; 2 Faculty of Medicine, University of Ottawa, Ottawa, ON, Canada

**Keywords:** Bullous pemphigoid, imiquimod, actinic keratoses

## Abstract

A 72-year-old man treated with 3.5% imiquimod cream for scalp actinic keratoses developed the usual crusted and erosive reaction but developed bullae on the scalp, as well as the limbs and torso after several weeks into treatment. Biopsy confirmed bullous pemphigoid. He was treated with clobetasol ointment, prednisone and methotrexate, with eventual disease control. He had a severe disease course. Bullous pemphigoid is usually idiopathic, but can be induced by skin trauma, as well as by several medications; this is the first report of imiquimod as a trigger. Imiquimod is a toll-like receptor 7 agonist that induces cellular apoptosis and recruits pro-inflammatory cytokines including tumour necrosis factor-alpha and interferon-alpha, which have been implicated in autoimmunity. This case highlights an unusual but severe adverse effect from topical imiquimod.

## Introduction

Bullous pemphigoid (BP) is the most common acquired immunobullous disorder. While most cases are idiopathic, some are associated with medications, notably furosemide.^1^ Other putative triggers include skin wounding, as from burns or excoriations.^2^ Herein, we present a case of BP associated with imiquimod.

## Case report

A 72-year-old male with type I skin was prescribed imiquimod cream 3.5% for scalp actinic keratoses (AKs). He applied the product once nightly for 2 weeks, and then took a 2-week break before starting the second 2-week treatment. He developed several painful crusts on the scalp from imiquimod as expected, but otherwise did not have any other side effects. A few days after starting the second treatment, he developed pruritic and painful blisters first on the scalp and then the hands, thighs, and abdomen, as well as in the mouth. He was initially treated with permethrin cream for suspected bullous scabies but did not show any improvement and was referred to dermatology. His active medications included amlodipine, lisinopril, atorvastatin, and escitalopram.

**Figure 1.**
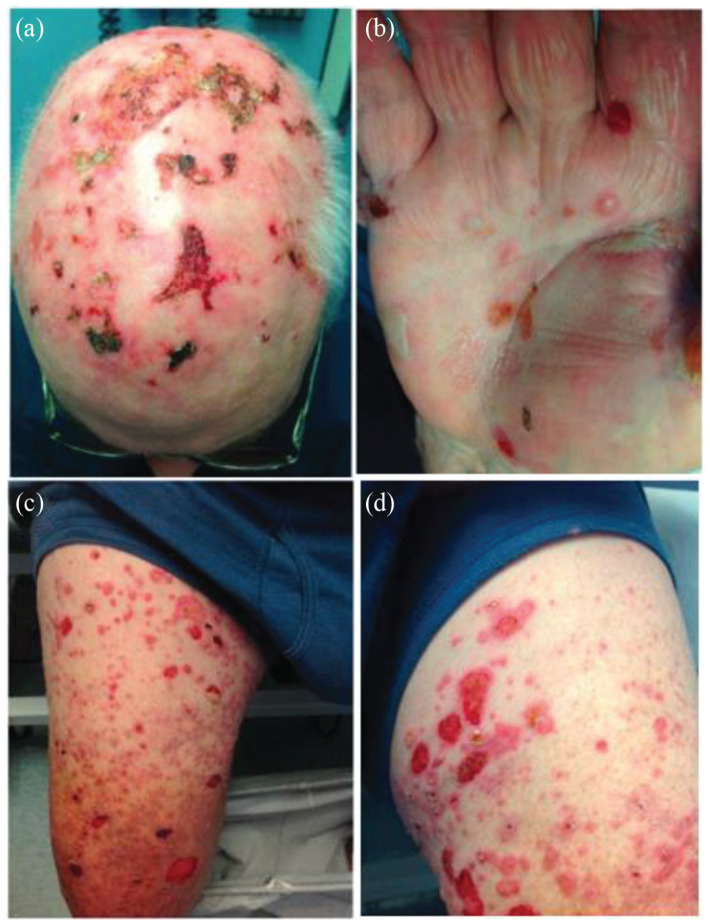
Numerous well-demarcated, hemorrhagic, crusted plaques, with erosions, vesicles, and bullae on the scalp (a) palm (b) and thighs (c & d).

On examination, there were crusts on the bald scalp, consistent with an imiquimod reaction to AKs. On the palms, dorsal hands, thighs, and abdomen, there were tense bullae on erythematous bases, with some lesions having a targetoid appearance (Figure 1). There were several ulcers on the labial and buccal mucosa. A diagnosis of erythema multiforme was considered, with BP as a differential diagnosis. Skin biopsies for Hematoxylin & Eosin, and direct immunofluorescence were performed and showed classic features of BP.

Imiquimod was discontinued. He was treated with prednisone 50 mg/day PO, tetracycline 500 mg PO BID, and clobetasol ointment. A short course of hydromorphone was required for pain control. His lesions improved gradually, and prednisone was tapered over several months to 5 mg/day PO. He continued to develop intermittent small bullae on the scalp, hands, and knees, which healed rapidly with the use of clobetasol ointment. Unfortunately, he developed type II diabetes secondary to corticosteroids, necessitating empagliflozin and metformin. Risedronate was added for bone protection. When prednisone was tapered to 2.5 mg PO alternate days, he developed a flare, and methotrexate 15 mg/week PO was added. Prednisone was discontinued, and he remained well controlled on methotrexate monotherapy for 5 years, and the dose was recently tapered to 7.5 mg/week PO.

## Discussion

To our knowledge, this is the first reported case of BP onset associated with recent imiquimod use. In drug-induced BP, the temporal relationship between the initiation of the drug culprit and onset of BP can range from weeks to several months.^1^ In the present case, although the patient’s usual medications, amlodipine, escitalopram, and lisinopril, have all been independently implicated in drug-induced BP, these medications were started over 15 months prior to BP onset flare, without preceding dosing changes.^1^ The acute onset of BP noted several weeks following initiation of imiquimod therapy is suggestive for a causal drug-induced phenomenon. Although there are no prior reports of imiquimod-induced BP, reports have identified pemphigus-type skin reactions confirmed with direct immunofluorescence, following short- and long-term use of imiquimod.^3–5^

The pathogenesis of BP is complex and involves the production of IgG autoantibodies against hemidesmosomal proteins BP180 and BP230.^9^ The disruption of the balance between autoreactive T helper (Th) and T regulatory cells as well as T-cell-independent activation of the toll-like receptor (TLR) system is thought to stimulate autoantibody production.^6^ In addition, the Th17 pro-inflammatory immune pathway can stimulate a Th2 response, resulting in the release of many pro-inflammatory cytokines that contribute to the immune dysregulation response in BP.^6^

Imiquimod is a TLR agonist that induces cellular apoptosis and recruits pro-inflammatory cytokines, namely interferons (IFNs), tumour necrosis factor-α (TNF-α), interleukin (IL)-1, IL-6, IL-10, and IL-12.^3^ It is plausible that imiquimod may result in an immunomodulatory shift or modification of T-cell and cytokine profiles implicated in the disease process of BP. This notion is supported by murine models demonstrating that topical imiquimod increases autoreactive T-cells.^7^ Human studies have identified increased levels of IFN-γ, TNF-α, IL-1, and IL-6 in blister fluid of patients with BP, and serum IL-1 and TNF-α levels are correlated with increased disease severity.^8^ In addition, IFN therapy is known to induce autoimmune disorders and long-term exposure to IFN-α is associated with the development of epithelial autoantibodies.^5^

Various physical agents are putative triggers for BP, including mechanical trauma, radiotherapy, burns, and physical therapies such as ultraviolet (UV) radiation or psoralen plus UV-A therapy.^^2^,^9–11^^ It is possible that imiquimod may trigger BP by causing local epithelial disruption leading to a systemic immune response. This is supported in the present case by the initial appearance of blisters on the scalp, the localized area to which imiquimod was first applied, and the systemic reaction noted after subsequent re-application of imiquimod. Although the exact pathogenesis remains unclear, tissue destruction caused by these physical and chemical agents and subsequent tissue remodelling may induce an immune cascade leading to the immune dysregulation response in BP. In particular, the release of vascular endothelial growth factor during the tissue remodelling process increases vascular permeability which may increase the binding availability of anti-basement membrane antibodies that may already be present at sub-clinical titres in patients who are predisposed to developing BP.

As imiquimod is a commonly prescribed topical therapy for AKs, this case report highlights an unexpected serious adverse event which clinicians should be aware of. Older patients who are at greater risk of BP also represent a common age group with AKs. The development of blisters at treatment sites and beyond should prompt immediate withdrawal of imiquimod therapy as well as biopsy to exclude BP.
